# The Cryopreservation of Medicinal and Ornamental Geophytes: Application and Challenges

**DOI:** 10.3390/plants12112143

**Published:** 2023-05-29

**Authors:** Soumaya El Merzougui, Carla Benelli, Rachida El Boullani, Mohammed Amine Serghini

**Affiliations:** 1 Laboratory of Biotechnology and Valorization of Natural Resources, Department of Biology, Faculty of Sciences, Ibn Zohr University, Agadir 8106, Morocco; soumaya.merzougui@gmail.com (S.E.M.); rachidaelboullani@gmail.com (R.E.B.); m.serghini@uiz.ac.ma (M.A.S.); 2 Institute of BioEconomy, National Research Council (CNR/IBE), Sesto Fiorentino, 50019 Florence, Italy

**Keywords:** long-term conservation, plant germplasm, bulbous plant, pollen, shoot tip, buds

## Abstract

Nowadays, plant genetic resources are often at risk of loss and destruction. Geophytes are herbaceous or perennial species that are annually renewed by bulbs, rhizomes, tuberous roots, or tubers. They are often subject to overexploitation, which, combined with other biotic and abiotic stresses, can make these plants more vulnerable to a decline in their diffusion. As a result, multiple endeavors have been undertaken to establish better conservation strategies. Plant cryopreservation at ultra-low temperatures in liquid nitrogen (−196 °C) has proven to be an effective, long-term, low-cost, and suitable conservation method for many plant species. Over the last two decades, major advances in cryobiology studies have enabled successful explants of multiple genera and types, including pollen, shoot tips, dormant buds, and zygotic and somatic embryos. This review provides an update on recent advances and developments in cryopreservation and its application to medicinal and ornamental geophytes. In addition, the review includes a brief summary of factors limiting the success of bulbous germplasm conservation. The critical analysis underpinning this review will benefit biologists and cryobiologists in their further studies on the optimization of geophyte cryopreservation protocols and will support a more complete and wider application of knowledge in this area.

## 1. Introduction

The plant germplasm represents not only a vital genetic resource but also has great ecological, biological, therapeutic, social, economic, spiritual, and aesthetic significance and potential. Unfortunately, dramatic biodiversity loss has been recorded due to multiple factors. The main causes of this biodiversity loss are climatic changes, overexploitation, and the unreasonable collection of wild germplasm due to its enormous demand and value. Moreover, the degradation of natural habitats is caused by urban development along with the invasion of exotic species, pests, and diseases [1,2]. The loss of plant biodiversity has an impact not only on ecosystem performance but also on human livelihoods and food security. Over the last few years, great efforts and strategies have been employed for germplasm conservation.

The conservation of germplasm can be carried out by multiple strategies, including both in situ and ex situ conservation. The first approach is defined as the conservation of plants’ germplasm in their natural habitat or environment [3]; it allows the preservation of ecosystems, species, and the co-evolution of plants with environmental changes. Nonetheless, this approach is often insufficient to achieve a complete safeguard for endangered species [1]. Ex situ conservation methods consist of germplasm conservation outside its natural ecosystem under controlled conditions. This conservation can be carried out via botanical gardens, seed banking, DNA storage, pollen storage, or field gene banks, and it is used to preserve threatened and vulnerable species. Seed storage is the most widely implicated pillar of ex situ preservation. Nevertheless, this technique is not practicable for some recalcitrant seeds or vegetatively propagated species, such as bulbous plants.

The application of biotechnology has helped the progress of plant germplasm ex situ conservation, starting with in vitro propagation. In addition to the large-scale production of disease-free plants, this technique allows medium-term conservation of about 1 to 5 years. The success of in vitro conservation relies on several factors, including the type of explant, the medium, and the culture conditions. However, the most critical factor depends on the acclimatization of plants to ex vitro conditions [1]. A more radical method for long-term, safe, and cost-effective storage is cryopreservation. This technique has proven to be a promising conservation method, and it has been applied successfully to many species [4,5] but it often requires an optimized in vitro culture protocol for the species tested [6,7]. Indeed, most of the explants used in cryopreservation derive from in vitro conditions, and their regrowth after liquid nitrogen depends on this propagation technique [8]. Bulbous geophytes are plants with an underground storage structure, such as a corm, rhizome, or tuber, which helps them to survive and sustain themselves in extreme conditions. Bulbous plants mainly belong to the *Amaryllidaceae*, *Alliaceae*, *Hyacinthaceae*, *Liliaceae*, and *Iridaceae* families. Geophyte plants are cultured naturally in many countries worldwide and are exploited for their enormous benefits for ornamental (flower bulb), economic, or medicinal purposes. The preservation of geophyte plants by the traditional method has many limitations, such as being costly, labor-intensive, and having the risk of natural disasters. Hence, it is important to use alternative methods, such as cryopreservation.

In this review, details about different approaches to the cryopreservation technique and its recent advances are summarized. Moreover, up-to-date information and studies on the application of cryopreservation to several ornamental and medicinal geophytes are highlighted. The large family of *Orchidaceae* has not been included in this review as it will be the single subject of a subsequent paper.

## 2. History, Principles, and Fundamentals of Cryopreservation

The application of the plant cryopreservation method has its origins in the use of cryobiology on mammalian species [9]. The first successful plant species storage at ultra-low temperatures was reported by Sakai [10] on mulberry twigs. Since then, cryopreservation has been successfully applied to a wide variety of species [9,11]. Cryopreservation is usually undertaken using two main approaches: (1) classical techniques, referred to as controlled freezing or slow cooling, and (2) vitrification-based procedures.

The first procedure is based on the osmotic regulation of cell contents and freeze-induced dehydration of plant material using a slow cooling system, before cryogenic storage in liquid nitrogen (LN) [12,13,14]. This technique involves controlled cooling rates alone or in combination with colligative cryoprotectants [15,16]. In fact, slow cooling is the determinant factor of cell survival after conservation. With a slow decrease in temperature, ice crystals are forced to form in the extracellular solution.

The second approach is the vitrification procedure. The basis of this technology is the transition that water undergoes from its liquid state to an amorphous ‘glassy state’ [12,16,17]. In this transition, crystalline ice structures are not formed; therefore, ice injuries are avoided. The vitrification state is achieved by the dehydration of cells, either by the treatment of plant tissue in a mixture of highly concentrated cryoprotective solutions (penetrating and non-penetrating agents) or by physical desiccation and subsequent very rapid cooling by direct immersion in LN. This procedure is simple, easy to apply, and does not need specific apparatus for controlled cooling; it has high reproducibility, and it has been applied successfully to a wide range of species using several explant types [16,18]. Therefore, it is amenable and more widely used compared to slow-controlled systems [14].

**Figure 1:**
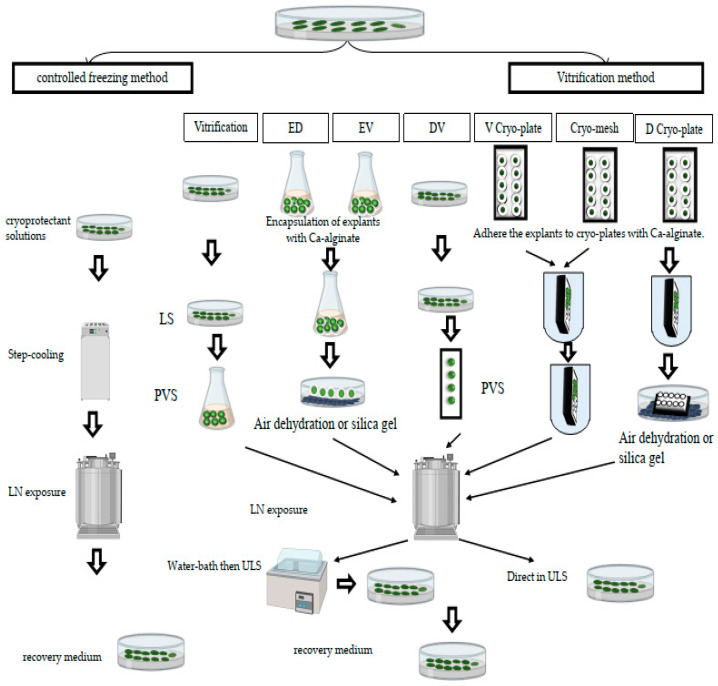
Cryopreservation techniques and the major steps in different procedures. ED: encapsulation–dehydration; EV: encapsulation–vitrification; DV: droplet vitrification; LS: loading solution; PVS: plant vitrification solution; LN: liquid nitrogen; ULS: unloading solution.

Different vitrification-based procedures can be mentioned: vitrification, encapsulation–dehydration, encapsulation–vitrification, droplet vitrification, and V- and D-cryoplate (Figure 1). Below is a short description of the procedures mentioned above.

### 2.1. Vitrification

Vitrification involves the treatment of plant explants with a highly concentrated cryoprotective mixture of penetrating and non-penetrating agents. The additive penetrates the cell cumulatively. Consequently, it increases the solute concentration, while the non-penetrating agent acts synergistically by withdrawing water osmotically. The use of these additive solutions increases the viscosity of the cytoplasm cell, avoiding ice formation.

The complete procedure includes several steps in order to improve the survival of tissue/explants after their immersion in LN.

(i) Pretreatment of explants: this step is important because it contributes to the dehydration tolerance of plants. It consists, generally, of explants’ treatment with a solution containing a high sucrose concentration for a short period (20–30 min): a loading solution (LS) [17].
(ii) Dehydration with complex mixtures of cryoprotectants, such as plant vitrification solutions (PVS2, PVS3). The most commonly employed mixture in cryopreservation is the PVS2 developed by Sakai et al. [19], which consists of 30% (*w*/*v*) glycerol, 15% (*w*/*v*) ethylene glycol, 15% (*w*/*v*) dimethyl sulfoxide (DMSO), and 0.4 M sucrose. Its exposure time to the explant can be a critical parameter in determining the possible toxic effect of the solution. The treatment period can vary (from 20 min to 200 min or more) depending on the type, size, and susceptibility of the explant. Moreover, most cryopreservation studies report that performing osmodehydration at 0 °C instead of 25 °C reduces the toxicity of PVS2 [7].
(iii) Rapid cooling by direct immersion in LN in order to promote the vitrification of internal solutes.
(iv) Rapid thawing of cryopreserved samples in a water bath at 40 °C for 1 min to 3 min. This step has to be completed very quickly to avoid devitrification, which would lead to ice crystal formation.
(v) Unloading treatment with a liquid medium containing 1.2 M sucrose or sorbitol (washing solution: ULS) is employed to remove the vitrification solution progressively and wash the cryopreserved explants.

### 2.2. Encapsulation–Dehydration and Encapsulation–Vitrification

The encapsulation–dehydration (ED) approach was developed by Fabre and Dereuddre [20]. This procedure is based on synthetic seed technology [21]. The dehydration process is promoted through water elimination by sterile airflow or silica gel [12,15,22]. In this method, the explant is first encapsulated in alginate-Ca beads, followed by treatment with a highly concentrated sucrose solution (0.7–1.5 M). The concentration is generally increased over several days in order to improve the intracellular solute concentration in the cells. The beads are then physically dehydrated under cabinet flow or with silica gel until an adequate moisture content is obtained (about 20–25%). Then, the beads are plunged into LN. The beads are thawed in a water bath at about 40 °C for 2–3 min. ED is laborious and time-consuming compared with the vitrification method, but it has the advantage of physical dehydration without cryoprotectant solution, which can be toxic for some explants, and overcomes the problems associated with the sensitivity of explants to PVS2 vitrification solution [22].

The encapsulation–vitrification (EV) technique is a combination of the two above-mentioned techniques: encapsulation–dehydration and vitrification. The samples are encapsulated in alginate-Ca beads, and then the beads are treated with a vitrification solution to dehydrate before direct immersion in LN [7,14,23]. The time used for dehydration is greatly decreased in EV compared to that of ED.

### 2.3. Droplet Vitrification

In standard vitrification, the explant takes place in a cryotube before immersion in LN, while in the droplet vitrification (DV) procedure, the explants are placed on aluminum foil strips inside a drop of cryoprotectant solution. This procedure was developed with potatoes by Mix-Wagner et al. [24], using 10% DMSO solution as the drops with shoot tips on aluminum foil strips and rapid freezing by direct immersion in LN. The advantages of this method are the specific contact between the cryoprotectant and the explant inside the droplets and the use of aluminum foil, a metal with efficient thermal conductivity that can ensure homogenous cooling by uniform temperature dispersion. DV was applied for banana cryopreservation by Panis et al. [25], and it resulted in a significant increase in the regrowth rate compared to the classical vitrification protocol. The DV method is the most widely applied cryopreservation method for various species, including herbaceous and woody plants [11,26]. The positive findings reported could be due to the more direct contact of explants with LN, facilitating both their rapid freezing and thawing [23,27,28].

### 2.4. V- and D-Cryoplate

V-cryoplate (VC) combines the two techniques, droplet vitrification and encapsulation–vitrification, in which encapsulation plays an essential role [29]. The VC method was developed by Yamamoto et al. [28] in order to facilitate large-scale cryobanks and reduce the damage and loss of samples during the cryopreservation procedure. This technique uses aluminum cryoplates with 10–12 small circular wells, where the explants are placed and which are then filled with a drop of alginate solution containing 2–3% (*w*/*v*) sodium alginate in a calcium-free MS basal medium [23,27]. They are then left for 15 min to achieve total polymerization with the calcium chloride solution. The samples are adhered to the cryoplate during the whole procedure. The explants are physically dehydrated in the air or with silica gel before immersion in LN and thawing in an adequate solution at room temperature [27].

D-cryoplate (DC) also employs aluminum cryoplates, as in the VC procedure. The main difference between VC and DC is the dehydration step. In this case, after encapsulation, the explants are treated with a loading solution, dehydrated with a vitrification solution on cryoplates, frozen in LN, and thawed at room temperature [23,27,30]. This technique was developed by Niino et al. [30] to avoid the damage that could occur at different stages, including physical damage to the explant during the cryopreservation steps, excessive osmotic stress, and the chemical toxicity of PVS treatment [23].

These techniques are the latest advancements in cryopreservation, and the advantages of both methods are that they are easy and simple techniques to perform and that the samples become sealed to the cryoplates. Thus, the possibility of injuring or losing samples during the cryopreservation procedure is low [23]. The adhesion of samples to the cryoplates depends on the following factors: the size of wells and explants, the volume of alginate gel, sucrose, and/or glycerol in the alginate solution, and the tension of the surface of the cryoplate [27,31].

A significant advantage is the high rate of survival and growth of sensitive cryopreserved shoots; also, in this case, the result might be due to the fact of overcoming the damages associated with PVS toxicity and explants’ sensitivity with respect to the classical vitrification procedure, where the explants are exposed, and to the high and rapid cooling and warming rates of treated materials, which improve recovery [23,30,32,33].

### 2.5. Cryo-Mesh

The cryo-mesh method was developed recently by Funnekotter et al. [34] for the successful cryopreservation of kangaroo paw (*Anigozanthos viridis*) shoot tips. The general procedure of this technique is similar to the VC method, and the main difference is the use of wire stainless steel mesh strips (cryo-mesh) instead of aluminum cryoplates.

## 3. Application of Cryopreservation to Some Medicinal and Ornamental Bulbous Plants

**Figure 2:**
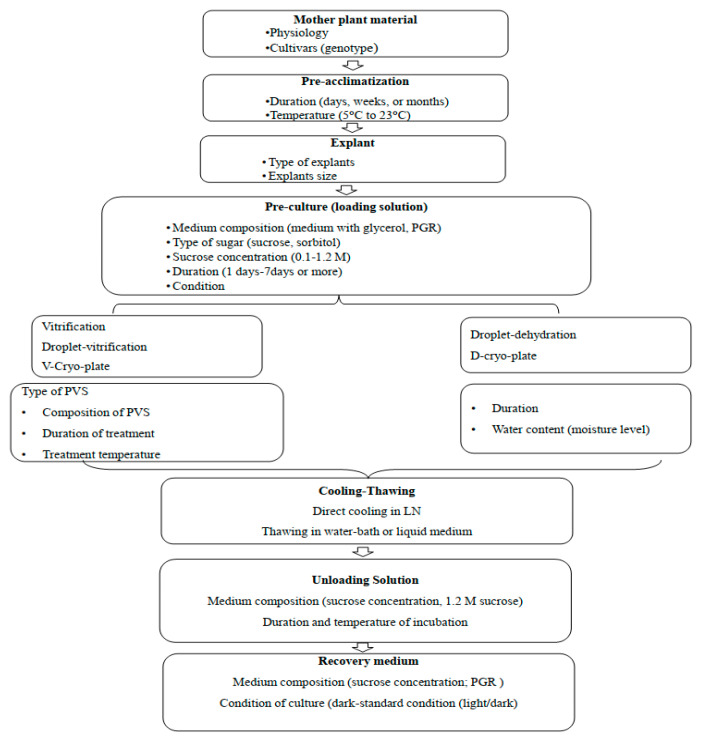
The major factors influencing the explants’ survival and regrowth in cryopreservation procedures.

The choice of cryopreservation methods adopted, as well as the processes applied, including preconditioning, preculturing, cryoprotectant treatments, storage in LN, rewarming, and the recovery of samples, are the main factors to successful conservation (Figure 2). All of these steps have vital impacts on achieving a higher survival and growth rate of cryopreserved explants, but it should be emphasized that the major issues related to bulbous species propagation, such as poor generation of new buds, low growth rate, and contamination, should be overcome first with an optimized in vitro propagation protocol.

In this section, the main factors investigated and reviewed on the cryopreservation of different geophytes germplasm are emphasized and are summarized in Table 1.

### 3.1. Amaryllidaceae

*Allium* genus—Earlier taxonomic classifications placed the *Allium genus* in the *Liliaceae* family, but recently it has been indicated to be more closely related to the *Amaryllidaceae* family [35,36]. Garlic is reported for its wide range of therapeutic effects, including antihyperglycemic, antibacterial, antifungal, anticancer, and cardioprotective effects [37]. Presently, several papers have reported that cryopreservation is the most appropriate and reliable tool for space- and cost-efficiency and for providing an unlimited storage period for this species [38,39,40] The first report of cryopreservation was of *Allium wakegi*, with seven cultivars successfully cryopreserved by vitrification. Just after, Niwata [41] reported the conservation of *A. sativum* apical meristems using the same method. Over time, many studies tested cryopreservation procedures for *Allium*, including vitrification [42,43,44,45,46,47], encapsulation–dehydration [48,49], droplet vitrification [46,50,51,52,53,54,55], and cryoplate methods [23]. A variety of explants were examined in these different cryopreservation procedures, including shoot tips [23,42,50,51,52,53,56,57,58], unripe inflorescences and bulbil primordia [54,55], pollen [59], and embryonic callus [47].

Several factors have been highlighted to influence the success of allium cryopreservation, among them the effects of post-harvest storage and storage duration, which are linked to the dormancy of bulbs after harvest. The pre-acclimatization or cold storage of garlic bulbs and their storage duration have also proved to affect the survival and regeneration of cryopreserved garlic material [56]. Further, Lynch et al. [48] have also reported the importance of the physiological state and the duration of the storage of bulbs on the survival and regrowth of cryopreserved stem discs dissected from bulbs by encapsulation–dehydration. They obtained the highest survival of encapsulated stem discs (75%) with bulbs maintained for a period of 5 months at 10 °C, while the regrowth was 55% after 4 months. The most critical factors for the recovery and growth of cryopreserved garlic shoot tips were the vitrification solution and dehydration duration, followed by the material (source and size), preculture duration, sucrose concentration in the preculture medium, warming velocity, and unloading duration.

The type of PVS to apply in garlic cryopreservation was another issue discussed in several papers. According to Makowska et al. [45], the average survival of the three garlic accessions tested was high using PVS3 (76–83%) in comparison with the PVS2 treatment (0–37%). Further, Kim et al. [60] demonstrated that higher survival and regeneration rates of explants (90.6% and 83.2%, respectively) were obtained using PVS3 for 150 min under the DV protocol compared to the original PVS2 for 30 min (79% and 66%). Wang et al. [51,52], applying the DV procedure, observed that PVS3 was suitable for shoot tips of *A. cepa*, a small-bulb onion featuring short growth periods and resistance to diseases; the optimal regrowth of explants (58%) was obtained with 3 h of osmodehydration with PVS3. Moreover, in the same study, they detected, by differential scanning calorimetry (DSC), a 1.8% content of freezable water in the shoot tips dehydrated with PVS2 and no freezable water in those treated with PVS3. Further validation of this state was provided by histological observations; indeed, they reported that many cells in the apical dome survived after LN using PVS3 dehydration, while with PVS2 treatment, fewer cells survived. On the contrary, Volk et al. [53] used PVS2 for 30 min at 0 °C under the vitrification procedure, with a range of regrowth cryopreserved shoot tips from 25% to 75%, depending on the garlic accessions. Tanaka et al. [23] optimized the V-cryoplate procedure for *A. chinense* shoot tips, obtaining the maximum regrowth (100%) with a precultured treatment for 3 days in MS with 0.3 M sucrose and dehydration with PVS2 for 20–40 min. Survival and regrowth depend also on the size of the bulbs and the size of the explants used. Generally, the explants derived from large bulbs were better than the small ones, as reported by Keller [46]; moreover, the explants with sizes of 0.5–1 mm and lengths of 1–2 mm were preferred. Indeed, if the size is too large, the cryoprotective substances have difficulty entering all parts, and the thermal tensions can cause tissue ruptures during cooling or thawing; on the contrary, if the explants are too small, the ability to regrow can be lost [43,46]. The success of cryopreservation depends on the size of the final explants. Moreover, the type of explants used can also improve the success of cryopreservation in garlic. Wang et al. [52], applying the same DV protocol, observed different regrowth percentages using in vitro shoot tips (58%), adventitious buds (72%), and shoot tips from field bulbs (32%). Post-culture also has a significant impact on post-shoot tip regrowth. Keller [46] proved that a high regrowth rate of non-cryopreserved explants (95%) was obtained after preculture and post-culture on 0.3 M sucrose, and a 17.5% rate of cryopreserved explants was obtained with 0.44 M and 0.8 M sucrose preculture and post-culture treatments, respectively. Moreover, the author demonstrated the effect of explant types and cryopreservation procedures on the regrowth; the highest post-regrowth value was obtained from bulbils (72% and 80%) and in vitro plantlets (13% and 20%) using the vitrification and droplet vitrification techniques, respectively. Further, Wang et al. [51] also proved that more than 50% regrowth was obtained post-culture with 0.3 M sucrose for 2 days. However, post-regrowth of the shoot can depend on the genotypes/cultivars, as reported in some studies [50,52,53,54].

More recently, Tanaka et al. [58] reported the successful cryopreservation of *A. chinense* G. Don shoot tips by D-cryoplate for future applications in a gene bank. The authors embedded the explants on cryoplates with Ca-alginate. Thereafter, the cryoplates were immersed in an osmoprotection solution containing 2.0 M glycerol and 1.0 M sucrose and maintained for 30 min at 25 °C, followed by dehydration for 30 to 120 min at 25 °C. The cryoplate with explants was stored at −80 °C and −196 °C (LN); a high level of regrowth was recorded at both ultra-low temperatures. The average regrowth rates of the cryopreserved shoot tips recorded were 95.3% at −80 °C and −94.0% at 196 °C.

Cryopreservation is also a suitable method for the long-term preservation of *Allium* pollen. Ganeshan et al. [61,62] reported, in the first protocol for *A. cepa* pollen, that pollen viability and fertility were not influenced after 360 days of cryopreservation. In another study by Senula and Keller [59], different accessions within 78 *Allium* species showed a high mean germination of cryopreserved pollen, about 78%. In particular, the post-germination rate was cultivar-dependent, and the highest level, around 60%, was obtained with *A. cepa*, while only 25% was obtained with *A. obliquum.* The main papers on *Allium* cryopreservation are reported in Table 1.

*Amaryllis* genus—Amaryllis is a flowering geophyte that belongs to the *Amaryllidaceae* family, which comprises two species: *Amaryllis belladonna*, known as the ‘belladonna lily’, the only species reported in cryopreservation studies, and *Amaryllis paradisicola Snijma* [63]. The *Amaryllis* species have been used over time in folk medicine and as ornamental flowers. The application of cryopreservation is particularly important for this species as it is characterized by recalcitrant seeds with a short conservation period [64]. The earliest cryopreservation research was performed on embryonic axes by Sershen et al. [65]. In this study, they assessed fifteen species of *Amaryllidaceae*. The embryos were excised from the seeds of mature fruits, flash-dried until reaching a water content in the range of 0.4 to 0.1 g g^−1^, and immersed in different cryoprotectant solutions (glycerol and sucrose alone or in combination). The embryo axes then underwent two cooling systems: (1) direct immersion of explants in liquid nitrogen or (2) slow cooling. The best post-thaw survival rate (~70%) was achieved in several accessions tested after the flash drying of embryos using glycerol as the cryoprotectant solution and rapid cooling, followed by thawing with direct immersion for 2 min in a preheated CaMg solution at 40 °C, and immersion of the cryopreserved embryos in a CaMg solution at 28 °C for 30 min for rehydration in dark conditions. The maximum survival rate (85%) was recorded in *A. belladonna*. This condition was also supported by CryoSEM analysis on zygotic explants [66], where glycerol allowed a less destructive densification of the zygotic tissues during desiccation, particularly in *A. belladonna*. In the same species, Berjak et al. [66] confirmed these results, highlighting that the major contributory factor in the post-viability and survival of embryos was the pre-conditioning, involving the cryoprotectant solution with glycerol. Indeed, the highest survival was observed with glycerol treatment, while no viability was observed after sucrose treatment [65,66,67]. In an additional specific study on *A. belladonna* zygotic embryos [66], the survival rate was improved to 100% by applying the same protocol as that of Sershen et al. [68]. The glycerol solution, in combination with partial drying, enhanced post-cryopreservation viability in recalcitrant zygotic embryos of *A. belladonna* by protecting the activities of some antioxidant enzymes during the steps of cryopreservation [68].

*Galanthus* genus—*Galanthus* spp., known as snowdrops, are herbaceous plants belonging to the *Amaryllidaceae* family and are grown naturally in Europe and the Middle East. This genus is well known for its alkaloid, flavonoid, and terpenoid content [69]. Cryobiology applications on *Galanthus* were first reported by Pawłowska [70] on somatic embryos by the encapsulation–dehydration technique. In this study, somatic embryos of *G. nivalis* L. and *G. elwesii* were encapsulated in MS medium enriched with 3% sodium alginate. After encapsulation, the explants were dehydrated by a quick method (in liquid media containing 0.75 M sucrose for 18 h) or by a gradual method (transfer into liquid media with increasing sucrose concentrations from 0.3 M to 1 M for 7 consecutive days). No post-regeneration was recorded after cryostorage, although the somatic embryos did not show browning. Later, Maślanka et al. [71] developed the droplet vitrification procedure, demonstrating successful cryopreservation of *G. elwesii* apical meristems. The explants were loaded into LS solution for 20 or 30 min at room temperature, then treated with PVS2 at 0 °C for 10, 20, and 30 min before plunging into LN. The thawing was performed with ULS 1.2 M sucrose in MS medium. In this work, the authors demonstrated that there is no significant effect of the duration of LS on the survival percentages, while the duration of PVS2 treatment has a strong effect on survival and regrowth. The high regrowth rates of 65.5% and 75% were obtained after 20 and 30 min of treatment, respectively, without statistical significance differences; the lowest regrowth rate (40%) was observed after 10 min of PVS2.

*Narcissus* genus—Narcissus is a perennial geophyte, widely spread in the Mediterranean basin and also in China and Japan. It is cultivated as a cut-flower and for its medicinal properties. Maślanka et al. [72] are the first and only authors to report on the PVS2-based droplet vitrification procedure for Narcissus L. ‘Carlton’ somatic embryos. The globular somatic embryos of different sizes, 1, 2, and 3 mm, were treated at room temperature for 20 min with LS. Thereafter, the explants were soaked in PVS2 for 10, 20, 30, 45, and 60 min at 0 °C. The somatic embryos were transferred in a droplet of PVS2 into a strip of sterile aluminum foil (2 cm × 0.5 cm), and the strips were plunged into LN. Later, rapid thawing was performed by quick soaking of the aluminum strips in ULS composed of 1.2 M sucrose in MS medium for 15 min at room temperature. The explants were placed into a semi-solid recovery medium composed of 1.2 M sucrose in MS medium for 1 day, then transferred to MS containing 30 g L^−1^ sucrose, 5 µM BAP, and 0.5 µM NAA in the dark. The main factors that may potentially influence the post-regrowth highlighted in this study were the effects of embryo size and PVS2 duration. The highest survival rates, 93.3% and 100%, were recorded using 2 mm and 3 mm somatic embryos, respectively, and applying the PVS2 treatment for more than 20 min. In the embryos with a size of 1 mm, the survival rate did not extend beyond 76.7%, but the growth rate (20%) was recorded only in these embryos after 60 min of PVS2, while no regeneration was achieved in large-size embryos.

### 3.2. Aracaceae

*Colocasia* genus—*Colocasia esculenta* (L.) Schott, known as taro, is an herbaceous plant belonging to the Aracaceae family. This plant is mainly cultivated in tropical and subtropical regions, and its corms are exported worldwide [73] for food purposes owing to their high starch, polysaccharide, and vitamin content and for medicinal value, including their antioxidant, antimetastatic, and anti-inflammatory properties, which occur as a result of secondary metabolites [74,75]. These nutritional, medicinal, and pharmaceutical properties make the taro corm a valuable plant genetic resource. However, a rapid decline of this plant in its natural habitat has been recorded due to overexploitation and its disease incidence caused by the pseudo-fungus *Phytophthora colocasiae*, leading to either stunting or failure to produce a corm. Thus, the development of long-term conservation is heavily needed [76]. During the last 20 years, several studies on cryobiology applications of the taro species have been reported. The first application was mentioned on embryogenic callus by Shimonishi et al. [77] using a slow freezing method. Later, other cryopreservation techniques were applied, including vitrification [78,79], droplet vitrification [80,81], and encapsulation–dehydration [76,82] on various explants, such as shoot tips [76,78,79,81,82], apical meristems [80], pollen [83], and axillary buds [78]. *C. esculenta* is a species characterized by its asynchronous flowering. To overcome this issue, an experiment was conducted to preserve the pollen in liquid nitrogen and then use the cryostored pollen for hybridization of taro [83]. The pollen was maintained in LN for different time intervals, ranging from 1 week to 2 months. After each conservation period, the viability and germination capacity were evaluated. No significant differences were recorded between fresh pollen and cryopreserved pollen from various taro accessions.

The main factors that influence the vitrification and encapsulation–dehydration procedures of taro shoot tips were discussed by Thinh and Takagi. [79]. In particular, they compared both procedures and found that vitrification was superior, showing higher levels of survival (75–100%; range of different taro cultivars), requiring a shorter time for the procedure, and not leading to callus formation from the cryopreserved shoot tip. Moreover, the small shoot tips were much more suitable for cryopreservation than the larger ones. Taro explants appear to have a low PVS2 sensitivity; this result was also confirmed by Thinh and Takagi [79], with the highest regeneration rate (94.7%) using the vitrification method rather than encapsulation/dehydration (85.5%). Further studies have noted more than 70% recovery recorded over a range of 20–40 min of PVS2 treatment [78,81].

The effect of sucrose duration and concentration on preculture treatment was observed in cryopreserved taro shoot tips by encapsulation–dehydration [76]. In this research, a survival rate of 63.9% was recorded in a medium containing 0.75 M sucrose for 2 days; the increase in concentration (more than 1 M) and duration of preculture (3–4 days) negatively influenced the recovery of the shoot tips. After sucrose preculture, the encapsulated explants were dehydrated to an 18–19% moisture content with silica gel or under laminar flow and were directly plunged into LN. Rapid thawing was performed in a 40 °C water bath for 3 min. Acedo et al. [82] also proved that a high sucrose concentration affects the viability of explants, with no sign of regrowth after LN treatment.

Taro apical meristems’ cryopreservation by droplet vitrification was reported by Noor Camellia et al. [80]. The authors highlighted a suitable condition for a high survival rate, recorded at 77.8%, with LS (1.5 M glycerol + 5% DMSO + 0.4 M sucrose) for 20 min, followed by 10 min of osmodehydration with PVS3. The regeneration of cryopreserved meristems exposed to PVS3 for more than 10 min was stunted, and they often died after four weeks. The ULS, consisting of 1.2 M sucrose for 15 min, was also defined as a key factor for the post-regrowth of cryopreserved explants.

The application of droplet vitrification on eighteen taro cultivars from the Asia-Pacific region showed a high range of regeneration rates, from 75 to 100% [81], using PVS2 at 0 °C with an optimal exposure range (20–40 min), and was equally applicable among the different genotypes assessed.

Different findings for taro were reported with the application of various cryogenic techniques, but regardless of them, the survival or regrowth of different cultivars of the same species can be influenced by genotypic dependence. For example, Thinh and Takagi [79] had lower taro cryopreservation success with var. *esculenta* than var. *antiquorum*. Moreover, Sant et al. [81], applying the vitrification method to different taro cultivars, obtained a satisfactory rate of regeneration only in three out of the eight cultivars investigated; the other ones showed low regeneration in the range of 21–30%.

### 3.3. Asparagaceae Family

*Asparagus* genus—The *Asparagaceae* family comprises about 150 species dispersed throughout the tropical and subtropical regions and persisting up to 1500 m elevation. Several studies have reported long-term conservation of this genus by cryopreservation applications, but only on the *Asparagus officinalis* species. *A. officinalis* is a perennial herb cultivated in more than 60 countries worldwide; its cultivation is important due to its economic value and high nutritional, medicinal, and therapeutic value [84]. Kumu et al. [85] developed the first cryopreservation protocol using the controlled freezing technique for *A. officinalis* shoot tips with a high post-survival rate of up to 100%. Other cryopreservation approaches have been applied, including controlled freezing [86,87,88], vitrification [86,89,90], desiccation–vitrification [91,92], droplet vitrification [93], and encapsulation–dehydration [94]. In these protocols, a variety of explants were tested, comprising buds [86,91,92], embryogenic cells [89,90,95] shoot tips [85,87,93], and rhizome buds [94]. Overall, the survival and regrowth percentages of cryopreserved explants ranged from 63% to 90% and 70% to 80, respectively, using different techniques. In the literature, several studies clarify contributing factors to the post-survival and regrowth rates after freezing of *A. officinalis*. Firstly, the preculture step in a medium supplemented with a high sugar concentration (0.2–0.7 M) for 1 to 2 days is crucial. During the preculture, the amount of water content decreases and, meanwhile, the concentration of sugar and protein increases [95]. This step is effective for improving the freezing tolerance and survival rate of the shoot tips. Kumu et al. [85] reported that 2 days of preculture in medium with 4% DMSO, followed by treatment with 16% DMSO solution, were also effective for the viability of samples (100% survival). The dried buds of asparagus pretreated for 2 days at 25 °C in culture medium with 0.7 M sucrose significantly increased their survival after immersing in LN (7% untreated; 63% treated) [91]. Suzuki et al. [87] confirmed that the preculture treatment increases the freezing resistance by ultrastructural studies that revealed the heterogeneous composition in terms of the viability of cells in dome tissues and reported a relationship between histological changes induced by preculture on sugar-rich media and an increase in freezing resistance in asparagus shoot tips. This finding also concerned embryogenic cell suspensions, as reported by Jitsuyama et al. [95]. The preculture of embryogenic cells for 2 days in a medium enriched with 0.8 M sucrose increased the freezing tolerance remarkably. These results were confirmed by electrolyte leakage. By contrast, other studies are in contradiction to these, for example, the 90% post-regeneration of bud clusters without any preculture or cold acclimatization treatment reported by Kohmura et al. [86]. Moreover, Nishizawa et al. [90] found a high embryogenic cell survival rate (86%) without pretreatment using a PVS3 solution (50% (*w*/*v*) glycerol and 50% (*w*/*v*) sucrose in water) for 20 min at 0 °C. Mix-Wagner et al. [93] applied the DV protocol to eight genotypes of asparagus without cold hardening, and obtained a survival rate ranging from 48% to 90% and a regrowth rate from 31% to 71%.

The moisture content of asparagus explants is another important factor; therefore, chemical and physical dehydration have been applied. Physical desiccation of buds has been applied by Uragami et al. [91,92]: buds were subjected to dehydration at 25 °C in sealed Petri dishes containing 15 g of dry silica gel for 24 h. A total of 63% of the cryopreserved buds remained alive after this treatment, with a 70% regeneration rate. A better regrowth rate of 84% after cryopreservation was obtained by Carmona-Martín et al. [94] by applying the encapsulation–dehydration method on rhizome buds: the encapsulated buds were subjected to desiccation over 24 h in jars containing 100 g of silica gel until a 35% moisture content, after which the explants were immersed for one hour in crushed ice at 0 °C, then for one hour at −20 °C in LN, and plunged directly in liquid nitrogen. Moreover, the cryopreserved plants showed a good rooting percentage (43%) and a high rate of acclimatization (95%). *A. officinalis* buds were successfully osmodehydrated by PVS2 treatment for 120 min at 0 °C, with the average number of shoots produced from each segment at 3.5 [86]. Cultured callus and somatic embryos of asparagus were treated using the vitrification procedure before immersion in LN by PVS1 containing 22% glycerol, 15% ethylene glycol, 15% propylene glycol, and 7% DMS0 in MS medium with 0.5 M sorbitol [89] or by PVS3 consisting of 50% glycerol and 50% sucrose [90]. The latter study reported the survival of cryopreserved asparagus embryogenic cells by different cryogenic protocols: conventional slow freezing, simple freezing, and vitrification. Among the protocols tested, vitrification was the best in terms of the survival rate.

### 3.4. Asteraceae Family

*Helianthus* genus—The Jerusalem artichoke (*Helianthus tuberosus* L.) is a perennial plant that is originally native to Native America and is cultivated for the direct consumption of the tuber. It was introduced to Europe in the 19th century and later spread to Japan as a folk remedy for diabetes [96]. Recently, it received renewed interest due to its economic, pharmaceutic, and medicinal values. Jerusalem artichoke germplasm collection and accession are conserved in field collections or in national gene banks as tissue culture in different countries, including in the Chinese Academy of Agricultural Sciences and the United States Department of Agriculture-Agricultural Research Service (USDA-ARS) [97]. However, field collection is labor-demanding and very costly. Therefore, cryopreservation is the ideal solution and can ensure the storage of plants for an indefinite period. Cryopreservation of the Jerusalem artichoke was reported by controlled freezing of the cell suspension, vitrification, and droplet vitrification on the shoot tip. Swan et al. [98] were the first to report *Helianthus tuberous* L cell suspension conservation using controlled freezing. Later, Harris et al. [99] indicated that the reduction in Tgase activity and a-tubulin tyrosination of the cryopreserved cell was related to a lack of post-thaw recovery when mannitol preculture treatment was applied. Volk and Richards [100] applied the vitrification method to cryopreserve shoot tips of nine Jerusalem artichoke cultivars, obtaining an average shoot regrowth of 34% after 15 min or 30 min of PVS2 treatment at 0 °C without significant differences in time exposure. More recently, Zhang et al. [97] developed an optimized droplet vitrification protocol for the shoot tips of four Jerusalem artichoke cultivars. In particular, they optimized protocols based on the following steps: preculture excised shoot tips in liquid MS medium with 0.4 M sucrose for 3 days, osmoprotection in loading solution for 30 min, treatment with PVS2 for 15 min at 0 °C, immersion in LN, and rapid thawing in MS liquid medium containing 1.2 M sucrose. Shoot tips of cv ‘Shudi’ showed the highest survival (93%) and regrowth (83%) rates. The survival and regrowth obtained from other cultivars tested an average of shoot tip survival and regrowth ranging from 44% to 72% and 37% to 53%, respectively.

*Smallanthus* genus—Yacon (*Smallanthus sonchifolius*) is a perennial plant of the Asteraceae family. It is native to the Andean region [101] and is currently widely cultivated in several countries for its highly nutritious properties and medicinal value. In particular, its tuberous roots have a high content of inulin-type fructo-oligosaccharides (FOS) [102]. Yacon plants are vegetatively propagated by tubers, thus their genetic variability is quite low [103]. This factor, combined with intensive agriculture practices and the broad selection of varieties for food purposes, suggests the need to adopt new conservation strategies in order to maintain the variability of this species [104]. The only known study of cryopreservation of the yacon is on the apical buds by the droplet vitrification method reported by Hammond et al. [104]. They used the apical buds excised from 2–3-week-old in vitro plantlets and precultured them in MS medium containing 0.3 M sucrose in dark conditions overnight, then treated them with LS for 20 min at room temperature. The study focused on the use of two plant vitrification solutions: PVS2 (15, 30, and 60 min) at 0 °C and PVS3 (30, 45, 60, and 75 min) at 22 °C using the DV procedure. Afterward, the cryopreserved buds were washed in an unloading liquid medium with 1.2 M sucrose for 15 min. This study reported no significant effect of LS in bud regrowth and no significant differences between PSV2 and PVS3 when they were used for 60 min. Moreover, they highlighted that hormone-free MS medium is optimal for the recovery of the post-cryopreserved buds. This protocol gave the maximum survival and regrowth at 87% and 90%, respectively. The optimal protocol developed was then applied to four clones of yacon, reporting a survival rate ranging from 79.7 to 94.1% and a regrowth rate of 66.3 to 75.4%.

### 3.5. Basellaceae Family

*Ullucus* genus—*Ullucus tuberosus* is a tuber crop, indigenous to the Andean region of South America. It is cultivated mainly for its edible tubers and economic value; this crop plays a significant role in the economies of the local communities of the Andean region [105]. However, a rapid decline in genetic diversity over the last decades has emerged as a result of the food selection of the Andean tuber variety, the intensive cultivation of the monocultural crop, and the marginalization of the species. Thus, advanced approaches for long-term conservation are heavily needed and should be complementary to traditional methods, such as field collections, as the risk of germplasm loss caused by climate events and unexpected diseases is highly possible [106]. Different procedures of cryopreservation, such as droplet vitrification [107,108], desiccation [108], V-cryoplate, and D-cryoplate [106], have been applied to the *Ullucus* genus, and, at present, the only explant type used is the shoot tip derived from in vitro culture.

The D-cryoplate method showed the highest shoot recovery rate with an average of 89.7% on the eleven lines of *Ullucus* tested [106], while the cryopreserved shoot tip regrowth using droplet vitrification was 52.5% [108]. Sánchez et al. [107], by applying the DV procedure, emphasized the effect of preculture and cryoprotectant time on the recovery of the cryopreserved shoot tips. The study found that continuous preculture treatment at 18 °C had a positive effect on explant recovery, and PVS2 treatment for 60 min had the highest values (survival rate of 38.8% and regrowth rate of 34.6%). A longer exposure period decreased shoot tip survival. Furthermore, low temperatures on mother plants compared with culturing at a constant temperature of 18 °C had no significant effect on the regeneration of cryopreserved shoot tips. Zamecnikova et al. [108], with respect to the DV protocol, highlighted the significant effect of pretreatment of the shoots with 2 M sucrose and the application of PVS3 treatment for 1.5 h to achieve a high regeneration rate (52.5%) compared to the use of desiccation on silica gel with just a 10% shoot tip regrowth rate. The results by Arizaga et al. [106] showed superiority in terms of regrowth using the D-cryoplate procedure (76.7%) compared to V-cryoplate (43.3); moreover, for further improvement of the D-cryoplate procedure, they highlighted the positive effect of cold hardening for 3–4 weeks, the addition of 0.4 M sucrose in the polymerization solutions (sodium alginate and CaCl_2_), a time exposure of LS for 30 min, and a dehydration time under an air laminar flow cabinet for 60 min at 25 °C. This optimal D-cryoplate procedure gave the maximum regrowth of 96.7% in three lines of *U. tuberosus*.

### 3.6. Berberidaceae Family

*Podophyllum* genus—Podophyllum is an herbaceous, rhizomatous plant with great medicinal value. Its medicinal properties are due to its high aryltetralin lignan content [109]. The only application of cryopreservation carried out on *Podophyllum hexandrum* seeds was reported by Kushwaha et al. [110]. In their study, 14 accessions of mature *P. hexandrum* seeds were extracted from ripe red berries and desiccated using a desiccator containing CaCl_2_. Four moisture content levels were tested at 5, 10, 20, and 50% for the cryopreservation experiment. Thereafter, the seeds were directly immersed in LN. The highest survival rate was obtained with a 5% and 10% moisture content over a period of 24 months of conservation in LN, with a mean seed germination rate of 89%. While, with 20% moisture content, a good germination rate was recorded until 10 days of cryopreservation, beyond this period the germination stopped. No seed survival was observed with a 50% seed moisture content.

### 3.7. Colchicaceae Family

*Gloriosa* genus—*Gloriosa superba*, known as the flame lily, tiger lily, and glory lily, is a perennial plant belonging to the *Colchicaceae* family. It is grown naturally in Africa and Southeast Asia in tropical and subtropical regions. This plant is mainly exploited for its high medicinal value [111], in particular, for colchicine production. This species has been listed as endangered due to overharvesting, poor seed germination, and slow tuber multiplication. Thus, a well-developed protocol for its propagation, rapid multiplication, and effective conservation methods are becoming a priority; biotechnological approaches including in vitro culture and cryopreservation methods for the long term are now available.

However, the only cryopreservation application for this species is described by Rajasekharan et al. [112] for pollen conservation. In their study, the pollen was collected in the flowering phase in the early morning to ensure a high viability rate for explants, the pollen grains were desiccated to a moisture content of around 15–30% t before being plunged into liquid nitrogen. Cryostorage pollen was thawed at room temperature for 3–5 min, and viability was carried out with the hanging drop technique using Brewbaker and Kwack’s medium enriched with 10% sucrose solution. The fertility capacity was also evaluated compared to fresh pollen and cryopreserved pollen germination. This procedure was effective for boosting field collections and hybridization programs.

### 3.8. Iridaceae Family

*Crocus* genus—The crocus genus includes sterile geophytes with vegetative-grown systems; their propagation relies entirely on corm multiplication. This genus is vulnerable to a large range of pathogens, environmental stresses, and diseases. Conventional conservation methods, such as bulb storage, are not often effective for this germplasm. Cryopreservation offers a cost-effective and efficient preservation method for this genus. MalekZadeh et al. [113] were the first to describe the successful vitrification procedure on the shoot tips of *Crocus sativus*. They indicated that the size of the corm is a crucial factor in explant survival after storage in LN. The highest survival rate of the shoot tips (85.8%) was observed but without any perceivable activity when they used an explant of 2 mm × 4 mm in size. Later, successfully cryopreserved embryogenic calluses (EC) of *Crocus hyemalis* and *Crocus moabiticus* were obtained by the vitrification technique [114]. The authors reported the importance of the type and duration of LS treatment combined with PVS2 incubation. After an application of LS for 20 min and a treatment with four stepwise increases in the concentration of PVS2 (20, 40, 60, and 100%) for 5 min at each concentration, and direct immersion in LN of the ECs, the survival and regrowth rates were of 66.7–50% and 50–44.4%, respectively, for *C. moabiticus* and *C. hyemalis*. The application of encapsulation–vitrification described by Baghdadi et al. [115] considerably improved the EC cryopreservation of both *Crocus* species. The maximum survival of cryopreserved encapsulated EC treated with PVS2 solution for 20 min at 25 °C, was 75.0% and 55.6%, respectively, for *C. hyemalis* and *C. moabiticus*, while the regrowth rate was 66.7% for both species.

*Gladiolus* genus—*Gladiolus* is cultivated for its ornamental and medicinal purposes; it has excellent potential in floriculture and international flower markets. The first application of cryopreservation to Gladiolus cultivars was reported by Rajasekharan et al. [116] using a controlled freezing approach. Later on, simple desiccation with preservation at −80 °C [117] and droplet vitrification techniques were used. The two main explants used were the pollen [116,118] and the shoot tips [119]. Pollen grains have a vital role in the reproductive process of flowering plants. Their conservation is a tool to maintain genetic resources and improve breeding programs’ efficiency by overcoming physiological, seasonal, and geographical limitations [120]. The success of long-term conservation of pollen samples relies on many factors, such as the stage of pollen collection, the pretreatment of the samples, and the methodology applied [62]. Furthermore, post-thawing is yet another key factor that greatly influences the retrieval of stored pollen because it is related to pollen metabolism and the reactivation of post-cryopreserved metabolic processes [121]. *Gladiolus* pollen conservation was reported by Rajasekharan et al. [116]: in this study, five selected cultivars were tested, and the mean post-germination rate recorded was 52.8% compared to fresh pollen (without storage) at 58.1%. Of particular interest is that there was no decline in pollen viability between 1 and 10 years of cryogenic storage, as assessed in cv ‘Jowagenaar’.

For shoot tips’ long-term conservation, Joung et al. [119] was the first and only work that reported the successful cryopreservation of five *Gladiolus* genotypes. In this study, the shoot tips were excised from cormels and precultured in MS containing 2 mg/L kinetin and 3% sucrose in the dark at 4 °C for 16 h. The precultured shoot tips underwent LS treatment for 20 min, were transferred in drops on aluminum foil, and were treated with different PVS2 times before the immersion of the samples in LN. A rapid thawing was carried out in a liquid ULS (MS with 1.2 M sucrose) for 20 min at 25 °C. In this research, the key factors highlighted as influencing the post-regrowth of the vitrified shoot tips were the cormels’ diameter and the PVS2 incubation time. Concerning the first parameter, the shoot tips excised from cormels that were less than 1.0 cm in diameter resulted in better shoot regrowth. In addition, the maximum post-regrowth rate of 54% was recorded after 120 min of PVS2 treatment in cv ‘Peter Pan’, while the regrowth rate was only 15% for explants of one tested *Gladiolus* breeding line with PVS treatment of 15 min. This suggested the need to optimize conditions for each genotype.

*Iris* genus—To date, only a few cryopreservation studies have been performed for the *Iris* genera. In two species cryopreservation was reported, in *Iris nigricans* the method used was encapsulation–dehydration of somatic embryos (SE) [122], and in *Iris pumila*, shoot tips were preserved by the vitrification procedure [123,124]. The different key factors discussed in these studies were the precultured and PVS2 timings. The preculturing of *Iris nigricans* somatic embryos in a medium containing 0.75 M sucrose for 3 days at 22 °C, then at 30 °C for 1 day prior to freezing, was reported by Shibli. [122]. The loading solution treatment for 30 min at 25 °C for *Iris pumila* shoot tips was necessary for post-regeneration because no post-growth was observed when the loading solution was not applied [124]. In addition, the study of *Iris pumila* cryopreservation emphasized other factors that could influence the post-survival and regrowth of Iris, such as sample size. Shibli [122] indicated that sizes of 2–4 mm in somatic embryos had the maximum survival and regrowth rates (54% and 60%, respectively), higher than smaller (1–2 mm) or larger (4–6 mm) ones. In addition, for shoot tips, as cited by Jevremović et al. [124], a size of 2 mm showed important outcomes for successful regeneration. Moreover, when cold hardening is applied to shoot tips, these explants can survive after being exposed to PVS2 solution both at 0 and 25 °C. However, the highest tolerance to PVS2 was achieved at 0 °C with an osmodehydration time of 5–20 min; these conditions helped to increase tolerance to ultra-rapid storage in LN, showing high survival (65% for 15 min) and regrowth (55% for 20 min) rates [124]. The rooting of cryopreserved *I. pumila* shoots was achieved with success (90%) on hormone-free MS medium, and the clonal fidelity of the cryopreserved plants and control was also recognized [124].

### 3.9. Liliaceae Family

*Chlorophytum* genus—*Chlorophytum* is a large genus belonging to the Liliaceae family and is distributed in tropical and subtropical regions. This genus includes various perennial species that are well known for their horticultural, ornamental, and medicinal values. Recently, this genus has received more attention due to the recent discovery of its pharmacology characteristics, such as anticancer and immunomodulatory activities [125]. This increased attention has led to overexploitation and habitat destruction. Moreover, the overexploitation along with the low germination rate of these species are the main reasons for the plants’ declining distribution. Various genus members have recently been listed as critically endangered species, such as *Chlorophytum borivilianum*, a tropical species. Thus, cryopreservation could be considered the most promising method for protection. The first and only cryopreservation method for this species was developed on meristems of *Chlorophytum borivilianum* by the vitrification procedure by Chauhan et al. [126]. In this study, the in vitro shoots were pre-acclimated for two months in MS media with 12% sucrose: the meristem explants were excised and precultured in MS enriched with 12% sucrose and 50 mg/L ABA for 48 h at 25 °C. Good survival and regeneration levels were obtained at 66% and 33%, respectively, with 20 min in LS (13.7% sucrose and 18.4% glycerol) at 25 °C and 30 min of PVS2 treatment at 0 °C. The factors highlighted were the optimal duration of PVS2 treatment and the effect of the preculture on the medium with ABA. Indeed, the preculture of explants in MS supplemented with 50 mg/L ABA and 12% sucrose had a significant role in the post-regrowth of the meristems, with better results than the preculture in a medium with 12% sucrose and 0.5 M glycerol. The authors asserted that the ABA effect could be associated with the freezing tolerance of protein synthesis.

*Fritillaria* genus—*Fritillaria* is a genus that includes about 100 to 140 species of bulbous plants distributed in the northern hemisphere and temperate regions. Fritillaria bulbs are known for their substantial medicinal and horticultural values [127]. The high demand and wide uses of the bulbs exert a strong pressure on the species and a consequential genetic erosion risk; hence, their conservation is required. To date, two cryobiology approaches have been used for the Fritillaria genus: the controlled freezing of *F. thunbergii* callus and pollen and the vitrification of *F. anhuiensis* shoot tips and *F. cirrhosa* callus. The key factors highlighted for the successful cryopreservation of *Fritillaria* are the explant age and desiccation level. Indeed, Su [128] reported that the highest viability (56.4%) was obtained using pollen from a flowering day with a 20% water content. Moreover, other experiments proved that the precultured period, the concentration of the cryoprotectants, and the duration of osmodehydration by PVS2 treatment need to be controlled. Zhu et al. [129] were the first to report the use of the vitrification technique for *F. anhuiensis* shoot tip cryopreservation. In their study, a high survival rate of 79.9% was reported after a 3-day preculture of the shoot tips in MS medium enriched with 0.4 M sucrose, then 20 min of 60% PSV2 treatment at 25 °C, and a step in ice-cooled vitrification solution for 60 min before rapid cooling in LN. Further, according to Wang et al. [130], an 80% survival rate of *F. cirrhosa* callus was obtained after 6 days in MS enriched with 30 g/L sucrose plus 5% DMSO, followed by a treatment solution composed of 60% PVS2 for 25 min, then osmodehydration in 100% PVS2 for 60 min at 2 °C. A longer pretreatment duration of 9 days in a medium supplemented with 10% DMSO containing 0.5 mol/L sorbitol was instead required to obtain a high survival rate (87.4%) of *F. cirrhosa* callus [131].

*Lilium* genus—Lilium is one of the most economically significant genera in many countries. It includes a wide number of ornamental species, which are required commercially for their attractive flowers, food properties, and medicinal functions [132,133]. Recently, several research papers uncovered its various medicinal and pharmacological properties, including antioxidant, antidepressant, and anti-inflammatory activity [134]. Several species, including *L. maculatum* var. bukosanense, *L. polyphyllum*, and *L. tsingtauense*, have been classified as endangered species [135].

Therefore, it is critical to preserve the *Lilium* genus, and cryopreservation can be the best choice for the conservation of lily germplasm resources as it is considered a cost-efficient preservation method for the long term. Moreover, Lilium genetic improvement programs are dependent on the provision of genetic resources, and Lilium cryobank germplasm can provide vegetal material useful for breeding programs. A detailed review reports on cryobiotechnologies applied to *Lilium* species [135].

The first application on shoot tips of *L. speciosum* was reported by Bouman and De Klerk [136] using a two-step freezing method, but they only obtained an 8% survival rate. Since then, different lilium explant types have been preserved with cryopreservation methods, including seeds [137,138,139,140,141,142], shoot tips [143,144,145,146,147,148,149,150,151], meristems [144,145,152], embryonic axes [137,138], and pollen [153]. Different cryopreservation procedures have been applied, such as droplet vitrification [147,148,149,151,152], encapsulation–dehydration [137,139,142], encapsulation–vitrification [138], vitrification [145,152,153], and desiccation [140]. Several factors have been highlighted in previous researches that had crucial effects on the post-survival and regeneration levels of the cryopreserved explants. The cold hardening of the shoot tips was identified as affecting the shoots post-cryopreservation in some studies. Indeed, the cold hardening of scale segments from 7 to 30 days gave good shoot formation (72%) from excised apical meristems with the vitrification procedure [145]. A similar result was also obtained after a cold hardening at 4 °C for 7 days of scale segments in droplet vitrification on five lily accessions with wider survival (57.7–89.5%) and regeneration (52.7–87.5%) ranges [149]. Urbaniec-Kiepura and Bach [154] compared the effect of two storage temperatures (5 °C or 20 °C) for *L. martagon* bulblets on the growth of their meristems after vitrification; the material stored at 20 °C in a medium containing 3% sucrose showed a survival rate of 65% and a regeneration rate of 87%. In contrast, in the study carried out by Yin et al. [147] using DV, the cryopreservation of shoot tips without any cold hardening was reported: six lily accessions were able to survive and regrow into shoots following DV, although this capacity varied among the genotypes tested. With the same protocol, Yin Z. et al. [148], from cryopreserved shoot tips of Lilium oriental hybrid ‘Siberia’, obtained embryogenic callus (with a 70% frequency) and shoot regrowth (90%).

The LS composition and treatment duration were optimized in several works using droplet vitrification. Yi [149] demonstrated that the high survival and regeneration rates, 89.5% and 87.5%, respectively, in Lilium species tested were obtained with an LS called LD1 (MS medium added of 35% PVS2) for 60 min at 23 °C, followed by PVS3 treatment for 240 min. Using this protocol, approximately 160 accessions of lily germplasm were preserved [143], with survival rates ranging from 58.3% to 66.4% and regeneration rates ranging from 54.3% to 58.5%. A recent study reported that pretreatment for 40 min with LS containing 35% of PVS3 was an efficient step in DV on the regrowth of cryopreserved adventitious buds of lily [155]. A further investigation reported by Yin et al. [151] obtained the highest regeneration frequency of shoots (more than 90%) with LS, containing 0.4 M sucrose, for 20 min at room temperature.

PVS2 duration was also a factor investigated in several works. A range of 3–7 h at 0 °C was applied by Wang et al. [148], with maximum survival and regrowth rates of 84% and 72%, respectively, obtained after 7 h of PVS2 treatment. Yin et al. [147,151] confirmed that the higher shoot regrowth rates (more than 90%) were recorded after 3–4 h of PVS2 treatment. Further, Yi et al. [143] obtained good survival (63.3%) and regrowth (56.7%) rates after 4–6 h of PVS2 treatment. In the case of using PVS3, Yi et al. [149] reported that the highest shoot survival and regeneration rates (89.5% and 87.5%) occurred after PVS3 treatment for 240 min at 23 °C for all of the cryopreserved lily accessions assessed. Bi et al. [146] evaluated the effect of the size of the shoot tips on the survival frequency of shoots after cryopreservation; the highest percentage (92.5%) was obtained using shoot tips of 1 mm, while with shoot tips of 2 or 3 mm, a slight survival decrease was observed but without significant effects. Xu et al. [150] instead showed that the rapid cooling of shoot tips can be performed in the vitrification technique by using 200 μL Eppendorf tubes instead of 1 ml cryotubes: the reduction in the volume of the vitrification solution can help to improve the heat exchange rate and to enhance cell survival after cryopreservation. Indeed, the size of the tube influenced the survival rate, which was recorded at 95.8% with an Eppendorf tube (200 μL) and 75.1% using a cryotube (1 mL).

As reported in Table 1, several cryopreservation procedures were applied to various explants of Lilium species. The different results obtained can be caused by any of several factors, including donor plant variety, status, and culture condition, as well as by different cultivars, even when using the same method. Chen et al. [152] compared cryopreservation by droplet vitrification and by vitrification procedures. They reported that DV is more efficient, leading to an increase in the survival and regeneration percentages of some lily cultivars compared to vitrification. The main difference between the two procedures is the freezing rate: the cooling rate in droplet vitrification is faster than that in vitrification.

For the lily seed cryostorage, Kaviani et al. [138] found a high germination rate of 75% after the pretreatment of the seed with sucrose and dehydration. A combination of cold hardening and pretreatment of the seed affected its germination capacity, as reported by Urbaniec-Kiepura and Bach [141]. In their study, a 100% germination rate was found for seeds that were stored at 15 °C for twenty-six weeks, treated in 0.75 M sucrose, followed by air desiccation (moisture content seed: 13.1%), and direct immersion in LN. The encapsulation of the seed could also reduce seed-freezing injuries [137,142]. The good germination rate of the seeds (50%) was obtained after encapsulation–dehydration and LN storage, while the non-encapsulated seeds did not survive [137]. In another study, the encapsulation–vitrification procedure determined only 10% of regrowth in cryopreserved seeds and embryogenic axes [137]. The main factor for post-germination was the moisture content of the seed, with the best range between 10% and 20% [137,139,141,142]. Interesting findings were obtained when different cryopreservation procedures were compared on seeds of *Lilium ledebourii* stored at 2–4 °C for 6 weeks [156]. Applying vitrification, encapsulation–dehydration, pretreatment with glycerol, and desiccation, the authors reported good germination after seed cryostorage without significant differences among the procedures tested, although the glycerol treatment and vitrification showed the highest seed germination after LN (97.5% and 97.4%, respectively). Moreover, the study highlighted the desiccation procedure (94.8%) as the best treatment because it does not need any chemical compounds.

As an explant for Lilium cryopreservation, pollen was also investigated [143] due to its importance in the breeding program. Pollen grains were desiccated at 4 °C for 2 h on silica gel (with a moisture content of about 7.3–7.7%), maintained at 20 °C for 20 min, and then plunged into LN. The germination percentage recorded after thawing was 51% for ‘Sorbonne’ lily accession and 48% for ‘Siberia’ accession after 420 days in LN. Further, Xu et al. [153] applied rapid cooling and vitrification procedures to the Lilium oriental hybrid, ‘Siberia’, reporting, respectively, 58.8% and 70.3% pollen viability. For vitrification, they used suspension pollen in an LS for 20 min at 25 °C and PVS2 treatment for 50 min on ice. The loading treatment with the dehydration step was critical for the post-survival of pollen; omitting one of those steps during the vitrification procedure reduced pollen viability significantly.

*Tulip* genus—Few studies on cryopreservation applications have been reported for the *Tulipa* L. genus [157,158]. The droplet vitrification procedure on apical meristems is the unique protocol that has been applied to this species. This approach could be a new prospect for establishing gene banks of ornamental bulbous plants, which are mainly propagated vegetatively and thus cannot be stored in seed banks. This study identified two factors that can impact the effectiveness of cryopreservation and bulb apical meristem recovery. The cold hardening of the bulbs and the PVS2 treatment time. The pre-storage of bulbs at 5 °C for 10 weeks before cryopreservation significantly improved their recovery rate after liquid nitrogen. Therefore, prior cold treatment of the bulb was fundamental for a better cryopreserved survival rate. Furthermore, the maximum survival and regrowth rates of cryopreserved apical meristems (100%) were recorded under 30 min or 60 min of PVS2 treatment [158].

### 3.10. Primulaceae Family

*Cyclamen* genus—The Cyclamen genus, classified in the *Primulaceae* family, includes 22 perennial species originating from the Mediterranean region [159]. Several species were cultivated for horticultural/ornamental interest, such as *C. purpurascens* Mill, and others for medicinal purposes due to their interesting biological properties [160]. To date, only two cryopreservation procedures have been successfully performed in cyclamen: controlled freezing and vitrification [161]. Winkelmann et al. [162], for the first time, reported the use of embryogenic suspension cultures of *C. persicum* for cryopreservation by the controlled–freezing method. They investigated the influence of the type and concentration of the cryoprotective solutions employed in the preculturing and pretreatment, in addition to the time of the pretreatment, on the cryopreserved explants’ regrowth. After testing the different concentrations of cryoprotectants (0.09, 0.2, 0.4, or 0.6 M sucrose or 0.4 M sorbitol) during the preculture step, the most successful combination was 0.6 M sucrose for preculture followed by 10% DMSO for pretreatment. These steps significantly improved the regrowth rate (75%) of embryonic callus culture. Furthermore, the optimal pretreatment time was 2–4 days. This tested protocol could be a useful tool for embryogenic cell lines of other cyclamen genotypes for storage in LN [163]. Later on, another study conducted by Izgu et al. [164] reported effective cryopreservation protocols for embryogenic callus in several cyclamen species (*C. cilicium*, *C. mirabile*, *C. parviflorum*, and *C. pseudibericum*) by the vitrification procedure. In this research, the authors, in addition to checking the genetic stability after cryopreservation, focused on factors influencing the post-recovery and regrowth of the cryopreserved explants, including the type of osmotic reagents used as cryoprotectants and the duration and temperature of the PVS2 solution. The highest regrowth rate of embryogenic callus without somaclonal variation was obtained using a pretreatment of 48 h at 4 °C in a medium with 0.5 M sucrose and a PVS2 treatment for 60 min at 0 °C (Izgu T; personal communication).

### 3.11. Ranunculaceae Family

*Aconitum* genus—The genus *Aconitum* belongs to the Ranunculaceae and is composed of approximately 400 species. Several plants in this genus are well known for their medicinal value due to their high secondary metabolite content, especially those in the alkaloid group. This genus has gained attention for the properties described above, which has led to its worldwide demand. *Aconitum heterophyllum*, known as ‘Ativisha’, is grown naturally in sub-alpine and alpine zones of the Himalayas. Its nontoxic tuberous roots are widely used in homeopathy and traditional Indian and Chinese medicine [165]. Due to its high value and demand, it has become increasingly threatened by illegal collection and marketing [166]. Therefore, adequate measures must be taken to conserve it. To our knowledge, the only application of cryopreservation was demonstrated by Kushwaha et al. [110] on *A. heterophyllum* seeds. In this study, the seeds were removed manually from dry follicles, then dried using a desiccant containing CaCl_2_ until a 5% or 10% of moisture content, followed by their direct immersion in LN. The germination rate of the desiccated, non-cryopreserved seeds recorded with both moisture contents tested (5% and 10%) was 90% and 89%, respectively. After one month of cryopreservation, the seeds of accession ‘Chamba’ with a 5% moisture content had a slight but significant decline in germination, while after 24 months, the germination rate remained constant (88%) without significant differences from that observed at one month.

### 3.12. Zingeberaceae Family

*Curcuma* genus—The genus Curcuma is well known for its multivarious uses as a spice, medicine, cosmetic, dye, flavoring, and ornament. Its name, Turmeric, is derived from the Latin word ‘*terra merita’* meaning meritorious land, which refers to the color of ground turmeric, which resembles a mineral pigment. The species that belong to the genus are currently threatened due to high anthropogenic interference and habitat destruction. The most widespread is *Curcuma domestica* Val. Syn. *Curcuma longa* is well known for its significant commercial and medicinal value. However, many species of the Curcuma genus have recently received more attention and generated worldwide commercial demand as ornamental plants, for example, *C. alismatifolia*, *C. amada*, *C. angustifolia*, *C. aromatica*, and *C. zedoaria* [167].

Curcuma species are characterized by low genetic variation as a result of sexual incompatibility due to their triploid nature. For this reason, a constant loss of genetic variability causes serious threats to extinction. Appropriate methods for medium- and long-term conservation are required to safeguard these species for future generations. The only cryopreservation study for these species is on *C. longa* by Islam [168], carried out with the DV procedure using in vitro axillary buds. In this study, the buds were treated with LS for 20 min, then incubated in three osmoprotection solutions, PVS [89], PVS2 [19], and Steponkus solution [169], for different time periods. The strength of the PVS2 solution compared to PVS2 0.60x and PVS2 0.80X was found to be more effective on the buds’ survival (53.3%), with an incubation time of 20 min. Moreover, the use of medium-sized buds (3–4 mm, a pretreatment on MS medium with 0.3 M sucrose) and thawing in a 1.2 M sucrose solution for 10 min led to the highest survival rate (80%) after cryopreservation.

*Kaempferia* genus—*Kaempferia galanga* L. is an endangered medicinal species cultivated in tropical Asia for its aromatic rhizome, which has a wide range of medicinal applications. Several uses of aromatic ginger for health benefits, food, and nutritional purposes are reported [170]. The rhizomes contain a high-value volatile oil, several alkaloids, starch, protein, aminoacids, minerals, and fatty matter, and the leaves and flowers contain flavonoids. Conventional propagation is by rhizomes, which remain dormant during drought periods and sprout in the spring. During maintenance in the field, the plants are under environmental pressures, such as diseases, pests, and extreme weather conditions. To avoid a loss of germplasm resources, in vitro propagation [171,172,173] and cryopreservation [174,175] as ex situ conservation techniques have been applied. Preetha et al. [174] reported the first cryopreservation study on *K. galanga*, assessing the effect of sucrose concentration during preculture, treatment with PVS2, and recovery medium on the cryopreservation of shoot tips. The optimized protocol included an overnight preculture treatment with 0.4 M sucrose, followed by dehydration with PVS2 for 20 min at 0 °C, immersion in LN, and thawing at 40 °C. The cryopreserved shoot tips were transferred on suitable medium recovery (MS + BA + GA3), showing survival and regrowth rates of 66.6% and 46.6%, respectively; the cryopreserved shoots also showed rooting after 30 days. In particular, the 20 min PVS2 treatment at 0 °C led to high shoot tip survival and regrowth rates. No significant difference was found after genetic analysis (RAPD). More recently, Preetha et al. [175] have applied encapsulation–dehydration to the shoot tips of *K. galanga.* The authors evaluated the effect of sucrose concentration and duration on the preculture treatment of encapsulated explants. The best result in terms of shoot recovery (around 50%) was obtained by preculturing with 0.3 M sucrose for 3 days together with 4 h of dehydration of beads under a laminar airflow cabinet, resulting in a bead moisture content of about 20–30%.

*Zingiber* genus—*Zingiber officinale* has been cultivated from time immemorial in India and China. It is used as an ingredient in many spice mixes in food preparation, and it is important for traditional Indian, Chinese, and Japanese medicine. Over 800 accessions of ginger germplasm are available in the National Conservatory for Ginger [176] at the Indian Institute of Spices Research (IISR). The main limitation involved in the conservation of the ginger germplasm relates to soil-borne diseases (*Pythium* spp.; *Pseudomonas solanacearum*). In addition, leaf fleck virus infection also raises several conservation issues. Considering that diseases are extremely difficult to control under field conditions, the ginger germplasm at IISR is actually preserved in specially made cement tubs under 50% shade as a nucleus gene bank to safeguard the purity of the germplasm. For the above reasons, it is important to apply a complementary strategy to protect genetic resources from diseases or other natural disasters. The first communication of the cryopreservation of ginger was reported by Geetha [177], using encapsulation–dehydration with a success rate of 40–50%. Later, Yamuna et al. [178] developed an efficient cryopreservation protocol for in vitro shoot buds of *Zingiber officinale* by comparing encapsulation–dehydration (ED), encapsulation–vitrification (EV), and vitrification (Vitr) procedures. The most effective cryopreservation procedure for ginger was obtained by vitrification, with an 80% shoot regrowth rate after cryopreservation. In this study, after preculturing the shoot buds in a medium with 0.3 M sucrose for 72 h, five different cryoprotectant mixtures and a PVS2 solution were applied. The latter was the most suitable cryoprotectant for shoot regrowth (80%) after liquid nitrogen and thawing in a water bath for 1 min. Moreover, the research highlighted the dehydration steps as the most fundamental factor for the successful application of ED and EV procedures. In ED, the regrowth of the cryopreserved ginger explants was greatly influenced by the moisture content of the precultured beads. Indeed, preculturing by increasing the sucrose concentration of the medium and dehydrating for 6 h under an air cabinet flow until a 21% bead moisture content, gave a recovery rate of 41% of the encapsulated ginger shoot buds. While, in EV, a treatment with 2 M glycerol and 1.6 M sucrose for 3 h at 25 àC showed a higher cryopreserved shoot regrowth rate (66%) when compared to the sucrose solution alone (24%).

**Table 1:** Cryopreservation of medicinal and ornamental geophytes. Best results are reported for each species (terminology and values are the same as mentioned by the authors).

Species	Explant Type	Cryopreservation Techniques	Survival/Regrowth (%)	References
***Amaryllidaceae***				
*Allium cepa*	Pollen	Des	R: 60	[59]
*Allium cepa*	Shoot tips	DV	R: 58	[52]
	Adventitious buds		R: 72	
	Shoot tips from bulbs		R: 32	
*Allium chinense*	Shoot tips	DV	R: 100	[23]
*Allium sativum*	Shoot apices from bulbs	Vitr	R: 90	[43]
*Allium sativum*	Shoot tips from bulbs	Vitr	R: 75	[53]
*Allium sativum*	Shoot tips	Vitr	R: 70	[44]
*Allium sativum*	Apices bulbs	DV	R: 100	[179]
*Allium sativum*	Immature involucres (bulbil primordia and floral buds)	DV	S: 90 R: 83	[60]
*Allium sativum*	Unripe inflorescences	DV	S: 79.9 R: 78.2	[54]
*Allium sativum*	Stem discs	ED	S: 75 R: 55	[48]
*Allium sativum*	Shoot apices	DV	S: 82.6 R: 75.9	[50]
*Allium sativum*	Shoot tips	D-cryoplate	R: 94	[58]
*Amaryllis belladonna*	Embryo axes	Des and cryoprotection	S: 85	[65]
*Amaryllis belladonna*	Zygotic embryos	Des and cryoprotection	S: 100	[66]
*Galanthus elwesii*	Apical meristems	DV	S: 96.7 R: 75.5	[71]
*Narcissus L.*	Somatic embryos	DV	S: 100	[72]
***Aracaceae***				
*Colocasia esculenta*	Embryogenic callus	CF	S: 75	[77]
*Colocasia esculenta*	Shoot tips	Vitr	S: ~80	[78]
*Colocasia esculenta*	Shoot tips	Vitr	S: 100	[79]
		ED	S: 85.5	
*Colocasia esculenta*	Embryogenic callus	EV	S: 60	[76]
*Colocasia esculenta*	Shoot tips	Vitr	S: 30	[180]
*Colocasia esculenta*	Shoot tips	DV	S: 100	[81]
*Colocasia esculenta*	Shoot tips	ED	S: 65	[76]
*Colocasia esculenta*	Apical meristems	DV	S: 77.8	[80]
*Colocasia esculenta*	Shoot tips	ED	S: 33	[82]
*Colocasia esculenta*	Pollen	Directly in LN	V: 86 * G: 16 *	[83]
***Asparagaceae***				
*Asparagus officinalis*	Shoot tips	CF	S: ~100	[85]
*Asparagus officinalis*	Somatic embryos	Vitr	S: 65	[89]
*Asparagus officinalis*	Cultured cells	Vitr	S: 48	[89]
*Asparagus officinalis*	Single node segments with axillary bud	Des	S: 63 R: 70	[91]
*Asparagus officinaIis*	Segment bud clusters	Vitr	R: ~90	[86]
*Asparagus officinalis*	Embryogenic suspension cells	Vitr	S: 86	[90]
*Asparagus officinalis*	Shoot tips	DV	S: 90 R: 71	[93]
*Asparagus officinalis*	Rhizome buds	ED	R: 84	[94]
***Asteraceae***				
*Helianthus tuberosus*	Shoot tips	Vitr	R: 34	[100]
*Helianthus tuberosus*	Shoot tips	DV	S: 93 R: 83	[97]
*Smallanthus sonchifolius*	Apical buds	DV	S: 90 R: 87	[104]
***Berberidaceae***				
*Podophyllum hexandrum*	Mature seeds	Des	G: 89	[110]
***Basellaceae***				
*Ullucus tuberosus*	Shoot tips	DV	S: 38.8 R: 34.6	[107]
*Ullucus tuberosus*	Shoot tips	DV	R: 52.5	[108]
		Desiccation	R: 10	
*Ullucus tuberosus*	Shoots tips	V-cryoplate	R: 43.3	[106]
		D-cryoplate	R: 96.7	
***Colchicaceae***				
*Gloriosa superba*	Pollen	Des	N.R.	[112]
***Iridaceae***				
*Crocus sativus*	Shoot tips	Vitr	S: 85.8	[113]
*Crocus hyemalis*	Embryogenic callus	Vitr	S: 66.7 R: 50.0	[114]
		EV	S: 75 R: 66.7	[115]
*Crocus moabiticus*	Embryogenic callus	Vitr	S: 50 R: 44.4	[114]
		EV	S: 55.6 R: 66.7	[115]
*Gladiolus* spp	Shoot tips	DV	R: 54.0	[119]
*Gladiolus*	Pollen	CF	G: 52.8	[116]
*Iris pumila*	Shoot tips	Vitr	S: 63 R: 55	[124]
*Iris nigricans*	Somatic embryos	ED	S: 60 R: 54	[122]
***Liliaceae***				
*Chlorophytum borivilianum*	Meristems	Vitr	S: 66 R: 33	[126]
*Fritillaria anhuiensis*	Shoot tips	Vitr	S: 79.9 R: 52.3	[129]
*Fritillaria cirrhosa*	Callus	Vitr	S: 80	[130]
*Fritillaria cirrhosa*	Callus	Vitr	S: 87.4	[131]
*Fritillaria thunbergii*	Pollen	CF	V: 56.4	[128]
*Fritillaria thunbergii*	Callus	CF	N.R.	[181]
*Lilium* spp	Apical meristems	Vitr	R: 85	[145]
*Lilium japonicum*	Scale segments with adventitious buds	ED	R: 90	[182]
*Lilium* spp	Shoot tips	DV	S: 89.5 R: 87.5	[149]
*Lilium* spp	Shoot tips	DV	S: 95 R: 87.5	[151]
*Lilium* oriental hybrid ‘Siberia’	Shoot tips	DV	R: 90	[148]
*Lilium* spp	Shoot meristems	Vitr	S: 90	[144]
*Lilium* oriental hybrid ‘Siberia	Shoot tips	DV	S: 92.5	[146]
*Lilium lancifolium*	Shoot tips	Vitr	S: 95	[150]
*Lilium* oriental hybrid ‘Siberia	Small leaf squares with adventitious bud	Vitr	S: 85 R: 72	[183]
*Lilium* spp	Apical meristems	DV	S: 83.8 R: 67.6	[152]
*Lilium martagon*	Meristem	DV	S: 65 R: 87	[154]
*Lilium* spp	Adventitious bulbs	DV	R: 65.7	[155]
*Lilium ledebourii*	Seeds	Des	G: 94.8	[156]
		Vitr	G: 97.4	
		Glycerol pretreatment	G: 97.5	
		ED	G: 69.4	
*Lilium ledebourii*	Seeds	Des	G: 100	[141]
*Lilium ledebourii*	Seeds	ED	G: 50	[139]
*Lilium ledebourii*	Seeds	Pre-growth dehydration	G: 75	[140]
*Lilium ledebourii*	Seed	EV	R: 10	[138]
	Embryonic axes		R: 10	
*Lilium* oriental hybrids	Pollen	Des	G: 51	[184]
*Lilium* oriental hybrid ‘Siberia’	Pollen	Rapid cooling	V: 58.8	[153]
		Vitr	V: 70.3	
*Tulipa tarda*	Apical meristems	DV	S: 90 R: 40	[157]
*Tulipa tarda*	Apical meristems	DV	R: 100	[158]
***Primulaceae***				
*Cyclamen persicum*	Embryogenic suspension cultures	CF	R: 75	[162]
*Cyclamen persicum*	Embryogenic callus	Vitr	R: 90	[164]
*C. cilicium*			R: 78	
*C. mirabile*			R: 80	
*C. parviflorum*			R: 70	
*C. pseudibericum*			R:75	
***Ranunculaceae***				
*Aconitum heterophyllum*	Mature seeds	Des	G: 88	[110]
***Zingeberaceae***				
*Curcuma longa*	Axillary buds	DV	S: 80	[168]
*Kaempferia galanga*	Shoot tips	Vitr	S: 66.7 R: 46.7	[174]
*Kaempferia galanga*	Shoot tips	ED	S: 56.7 R: 33.3	[174]
*Zingiber officinale*	Shoot buds	Vitr	R: 80	[178]

DV: droplet vitrification; Vitr: vitrification; EV: encapsulation–vitrification; ED: encapsulation–dehydration; CF: controlled freezing; Des: desiccation; LN: liquid nitrogen; G: germinability; V: viability; S: survival; R: regrowth; N.R.: not reported. * value after 72.

## 4. Conclusions

The World Health Organization (WHO) has reported that about 75% of the world’s population utilizes plant-derived medicines for their health and that around 21,000 plants are noted to have medicinal potential [185]. Despite the potential of new synthetic molecules, plants continue to be used as a major source of medicine throughout the world.

Many geophyte species are known for their pharmacological and therapeutic attributes because of their high content of natural bioactive compounds, such as carbohydrates, proteins, and mineral potentials, as well as for their ornamental and economic value. Given their potential value and due to their overexploitation combined with disease diffusion, environmental pollution, habitat fragmentation, climate change, urban expansion, and tourism, many plants from this group have been included in the list of Rare, Endangered, and Threatened plants. Therefore, the conservation and sustainable exploitation of plant genetic resources must be considered a priority if the future requirements of generations to come are to be met.

Furthermore, several geophytes are essential to the agricultural economies of many countries. As a source of carbohydrates for many people, they are a valuable basic food and can improve the livelihoods of the small farmers who cultivate them. Although geophytes have developed specialized organs to tolerate adverse environmental conditions, modified climatic conditions can deeply damage agricultural production, thereby affecting the lives and financial security of many people.

Biotechnological approaches to selecting, propagating, and preserving endangered genotypes are important tools in conservation activities. Cryopreservation can be a complementary method to conventional conservation to ensure that there is a back-up for the long-term storage of geophyte genetic resources. Some countries worldwide have developed cryobanks to preserve the main species of cultivated geophytes, such as cassava, potato, sweet potato, yam, and two of the species that are included in this review, garlic and taro. Currently, the practical application of the cryopreservation method is limited to the main economically important crops [186]. To encourage the further development and dissemination of the use of cryopreservation, it would appear advisable that simple and well-described protocols continue to evolve and adequate facilities and trained personnel be made available [187]. All these conditions will allow the development of new cryobanks in the future.

This review reported on the latest advances in cryopreservation and highlighted the key factors impacting the survival and regrowth of cryopreserved geophyte explants. We believe that this work will help researchers and the next generation of cryobiologists in their ongoing study of cryobiology.

## Data Availability

The data presented are available in all the publications cited in this review.
